# A New Aspect of the TrkB Signaling Pathway in Neural Plasticity

**DOI:** 10.2174/157015909790031210

**Published:** 2009-12

**Authors:** K Ohira, M Hayashi

**Affiliations:** 1 Division of Systems Medical Science, Institute for Comprehensive Medical Science, Fujita Health University, Toyoake, Aichi 470-1192, Japan; 2 Department of Cellular and Molecular Biology, Primate Research Institute, Kyoto University, Inuyama, Aichi 484-8506, Japan

**Keywords:** Brain-derived neurotrophic factor, development, intracellular signaling, morphology, neural plasticity, neuron-glia interaction, receptor dimerization, truncated TrkB-T1.

## Abstract

In the central nervous system (CNS), the expression of molecules is strictly regulated during development. Control of the spatiotemporal expression of molecules is a mechanism not only to construct the functional neuronal network but also to adjust the network in response to new information from outside of the individual, i.e., through learning and memory. Among the functional molecules in the CNS, one of the best-studied groups is the neurotrophins, which are nerve growth factor (NGF)-related gene family molecules. Neurotrophins include NGF, brain-derived neurotrophic factor (BDNF), neurotrophin 3 (NT-3), and NT-4/5 in the mammal. Among neurotrophins and their receptors, BDNF and tropomyosin-related kinases B (TrkB) are enriched in the CNS. In the CNS, the BDNF-TrkB signaling pathway fulfills a wide variety of functions throughout life, such as cell survival, migration, outgrowth of axons and dendrites, synaptogenesis, synaptic transmission, and remodeling of synapses. Although the same ligand and receptor, BDNF and TrkB, act in these various developmental events, we do not yet understand what kind of mechanism provokes the functional multiplicity of the BDNF-TrkB signaling pathway. In this review, we discuss the mechanism that elicits the variety of functions performed by the BDNF-TrkB signaling pathway in the CNS as a tool of pharmacological therapy.

## INTRODUCTION

The neurotrophins are the nerve growth factor (NGF)-related gene family molecules, including NGF, brain-derived neurotrophic factor (BDNF), neurotrophin-3 (NT-3), and NT-4/5. In the central nervous system (CNS), neurotrophins are expressed from the early embryonic stage to the adult stage and regulate a wide variety of functions, such as cell migration, outgrowth of neurites, synaptogenesis, cell survival and death, neuronal transmission, and synaptic plasticity [24,59,66,83,106,115,125]. These physiological functions of neurotrophins are induced by their specific receptors expressed on target cells. The neurotrophin receptors are categorized into two groups based on their binding affinities for neurotrophins [10,18]. One is the high-affinity tropomyosin-related kinase (Trk) receptor family, which includes TrkA, TrkB, and TrkC. NGF specifically recognizes TrkA, both BDNF and NT-4/5 are ligands for TrkB, and NT-3 binds to all Trks, although TrkC mediates the primary biological functions of NT-3. Another is low-affinity p75 neurotrophin receptor that is one of tumor necrosis factor (TNF) receptor family. This receptor can bind to all neurotrophins and enhance or suppress Trk signaling by the interaction between Trk and p75 [15], and transduce its own signals that regulate cell apoptosis or survive [28,110].

How do neurotrophins elicit their various functions? One way is by combining signal transducers [115]. Trks and p75 have many associated proteins that are the starting points of their signaling cascades [59,106,110,115]. These adaptors uniformly exist from early stages to adulthood and can transmit the signals of other growth factors, neurotransmitters, and hormones [31,80]. The associated proteins of all Trk receptors closely resemble each other [59,106,115], so the differences in adaptor protein combination can not explain not only the characteristic function of each neurotrophin but also developmental changes of neurotrophin functions.

Another possible mechanism by which neurotrophins elicit functions is the alternative splicing of the neurotrophin receptors. Generally, alternative splicing makes it possible to produce functionally distinct proteins that participate in diverse cellular processes, including differentiation and development [45,120]. Among Trk and p75 receptors, there are some alternative spliced forms [10,110]. Recent studies have revealed that splice variants of Trk receptors function as dominant negative forms [32,42,47,70,92], or they have distinct functions *via* their original signaling pathway [11,93, 96,98,109]. In this review, we focus on TrkB receptor, whose splice variants have been well studied, and discuss a new aspect of TrkB signaling for neural functions.

## STRUCTURES OF TRKB ISOFORMS

**Fig. (1). Schematic representation of TrkB isoforms.**
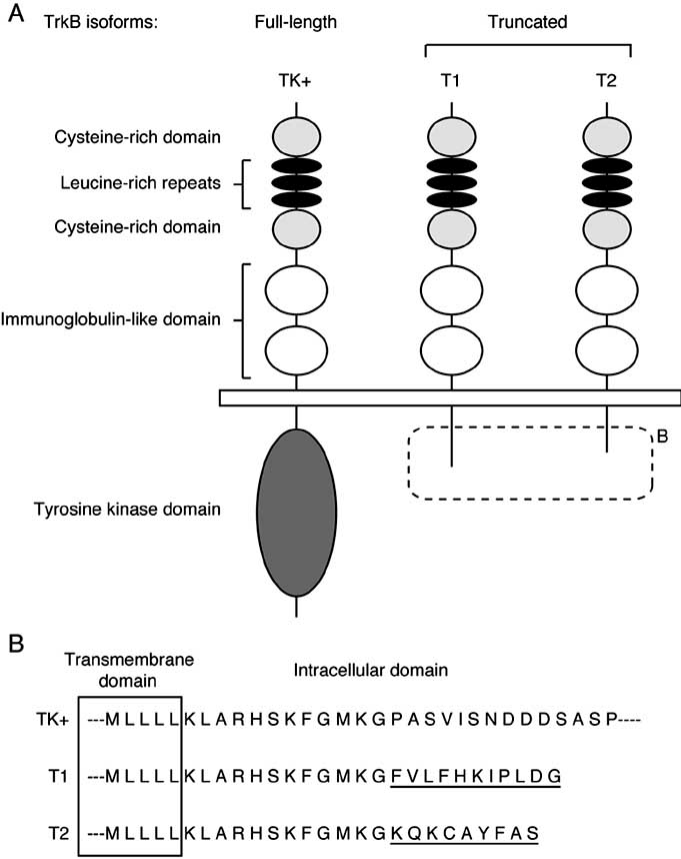
(**A**) Structures of TrkB isoforms are shown. The extracellular domain (i.e., cysteine-rich, leucine-rich, cysteine-rich, and two immunoglobulin-like domains), transmembrane domain, and initial 12 intracellular amino acid sequences are the same as those of T1 and T2. Truncated forms T1 and T2 possess 11 and 9 specific amino acid sequences, respectively. The dotted square indicates the specific sequences of truncated forms of TrkB, shown in B. (**B**) Comparison of intracellular amino acid sequences of TrkB isoforms. The parts shown in the square are transmembrane domains of TrkB isoforms. Specific amino acid sequences of T1 and T2 are underlined. In A and B, the T2-specific intracellular sequence is reported in the rat cerebellum.

Among neurotrophins and their Trk receptors, BDNF and TrkB are enriched in the CNS [66], and they play a pivotal role in neural plasticity during development and in adulthood [19]. TrkB is a single-pass transmembrane molecule. Alternative splicing of the TrkB pre-mRNA from the locus on DNA yields at least two isoforms (Fig. **1**) [86]. One is a full-length form of TrkB, which has the tyrosine kinase domain in the cytosolic region and is designated as TK+. The extracellular domain of TK+ possesses three tandem leucine-rich repeats flanked by two distinct cysteine-rich domains and two immunoglobulin-like domains, which are required for ligand binding, from the N-terminal [10]. Another is the tyrosine kinase lacking isoforms, TK-, which consists of two isoforms, T1 and T2. These truncated isoforms contain the same extracellular domain, transmembrane domain, and initial 12 intracellular amino acid sequences as TK+, but they have the specific C-terminal sequences (11 and 9 amino acid residues, respectively) [10]. Interestingly, the C-terminal sequence of T1 is completely conserved in mammals, such as mice, rats, and humans [67,86,118], suggesting that this sequence is essential for this isoform’s function. On the other hand, it remains unclear if T2 is expressed in mice and human, since the T2 sequence has been detected only in rats [67,74,86,118].

## EXPRESSION OF BDNF AND TRKB ISOFORMS IN THE CNS

BDNF is a secreted glycoprotein that is released from the pre- and postsynaptic terminals [3,36,37,51,71]. Importantly, the synthesis of BDNF is up-regulated in a neuronal activity-dependent manner. BDNF mRNA and protein are both detected in many CNS regions, such as the neocortex, amygdala, thalamus, hypothalamus, pituitary gland, and substantia nigra, suggesting an autocrine and paracrine mode of BDNF in those regions [57,132,133]. On the other hand, the synthetic and functional sites of BDNF are sometimes different. For example, the striatum contains BDNF protein but does not express BDNF mRNA. A previous study showed the anterograde transport of BDNF from the cortex to the striatum [5]. Among the CNS regions, hippocampal formation has been studied the most. In the rat, the dense positive structures of BDNF mRNA were observed in all regions of the hippocampus [33,62], but no immunoreactivity was found in the granule cell bodies or CA1 regions [132]. However, the mossy fiber layer was densely immunopositive for BDNF. One hypothesis is that BDNF mRNA is antero-gradely transported to the axons and/or dendrites of granule cells and CA1 pyramidal neurons, and locally translated to BDNF protein [19,64]. In contrast, both mRNA and protein of BDNF are expressed in all subregions of the monkey hippocampus [55,102,133]. In addition, the expression pattern of BDNF mRNA in the human hippocampus shows good similarity to that in the monkey hippocampus [129]. These differences in BDNF expression between rodents and primates may suggest different functions of BDNF in these species.

Previously, many studies of TrkB distribution focused on TK+ [6,20,131], because it is difficult to detect the immunoreactivity of T1. Since the T1 C-terminal is identical in mammals, as described above, the production of anti-T1 antibody is quite difficult. Recently, our group established the immunohistochemistry for T1, by the treatment with guanidine HCl (pH 11) that recovers the antigenicity of T1 [93-95,98,101]. The antibody of T1 recognizes the C-terminal 12 amino acid sequence that interacts with Rho GDI1, suggesting that the associated protein of T1 Rho GDI1 inhibits the interaction between T1 and anti-T1 antibody. The treatment with guanidine may dissociate the binding between T1 and Rho GDI1 or denature Rho GDI1, and then the antigenicity of T1 would be recovered.

As a result of previous immunohistochemical and *in situ* hybridization studies of TK+ and T1, researchers now know that both TK+ and T1 are widely distributed in all regions of the adult CNS, including the neocortex, cerebellum, hippocampus, amygdala, basal ganglia, septal region, thalamus, hypothalamus, midbrain, brainstem, and spinal cord [9,12, 20,41,53, 54,94,95,101,108,131]. On the other hand, western blot analysis with each antibody of TK+ and T1 has shown that the distributions of those molecules overlap considerably in almost all regions of the CNS in adulthood [2,41, 70,94,95,99,100,101].

## CELLULAR EXPRESSION OF BDNF AND TRKB ISOFORMS

In light of the expressions of TK+ and T1 at the cellular level, experiments have clarified that each expression pattern is considerably different from the other. In the neocortex of the adult rat, TK+ is detected in pyramidal neurons and GABAergic interneurons, whereas the expression of T1 is observed in not only neurons but also astrocytes [97,101]. Similar results were obtained at the TK+ and T1 mRNA level [9,12,40]. Northern blot analysis demonstrated that TK- is expressed in neurons, astrocytes, and oligodendrocytes, whereas TK+ transcript is only detected in neurons [40]. These results suggest that the interaction of TK+ and T1 may occur in neurons. In glias, T1 is a major isoform among TrkB subtypes and is involved in the function of glias.

## EXPRESSION CHANGES OF BDNF AND TRKB ISOFORMS DURING DEVELOPMENT

The expression of BDNF is observed beginning in the mid-stage of development in the mammal [60,89,90,114]. For example, in the developing cerebral cortex of the macaque monkey, which has an embryonic period of 165 days, BDNF mRNA was not detected before the 110th embryonic day (E110d), and the positive signals of BDNF were sparsely distributed in neocortical layers by E121d [60]. Also, at the protein level, BDNF content was at a low level at E120d, and then it gradually increased with the progress of development [89]. The level of BDNF protein in the monkey neocortex increased by 2-fold compared to the adult level at the early postnatal period, around postnatal 2 months (P2m), and decreased thereafter [89]. This increase in BDNF mRNA has also been reported in the human neonatal temporal cortex [129]. This developmental change of BDNF expression was also found in the rat occipital cortex, in which BDNF mRNA was at a low level by P10d, started to increase by 5-fold compared to the P10d value after the second postnatal week, and declined after P30d [114], indicating that this expression change of BDNF during development was conserved among mammals.

The developmental expression of TrkB isoforms exhibits a specific pattern [2,70,99]. TK+ is expressed in almost all regions of the CNS from the early developmental period, and its expression level is maintained into the adult stage. In contrast, the T1 expression in the forebrain, such as the neocortex, hippocampus, amygdala, olfactory bulb, striatum, and septum, is at a very low level by the early and middle developmental stages and increases markedly at the late developmental stage, with a high level of T1 expression maintained until the adult stage. Interestingly, the inflection points of both BDNF and T1 expression during development coincide well with the period of elimination of excessive axons and synaptogenesis [2,51,52,99].

## DEVELOPMENTAL CHANGE OF TRKB DIMERIZATION AND FUNCTIONS OF BDNF-TRKB SIGNALING

Neurotrophins exist *in vivo* as a non-covalently linked homodimeric protein, and the binding of neurotrophin to its receptor invokes the receptor dimer [10,18]. The dimerization of receptors induces autophosphorylation in the kinase domain of the cytosolic region of Trk receptors, followed by the activation of various signaling pathways, such as the Ras/MAP kinase, phospholipase C (PLC), and PI3 kinase pathways [24,59,66,106,115]. Thus, the dimerization of Trk receptor is very important as a starting point of intracellular signaling. It is interesting to know that the pattern of TrkB dimerization changes during development of the monkey neocortex [100].

**Fig. (2). TrkB signaling pathways.**
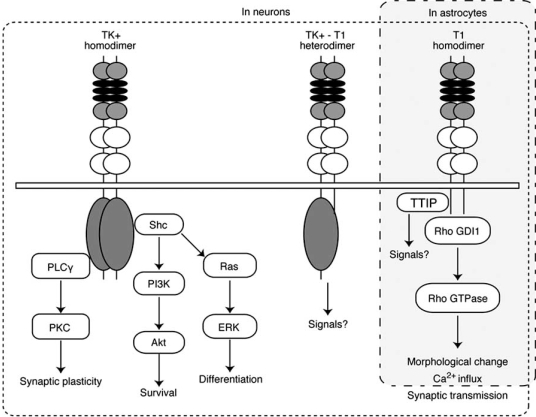
In the neuron, shown in a dotted square, BDNF induces three TrkB dimers: TK+ homodimer, TK+-T1 heterodimer, and T1 homodimer. The signaling cascade of TK+ homodimer has been well studied. Activation of PLCγ results in the activation of PKC, which promotes synaptic plasticity. Activation of Shc protein induces activation of the PI3K-Akt and Ras-MAP kinase signaling cascades, which regulate cell survival and differentiation, respectively. It is unclear whether TK+-T1 heterodimer can transduce the signals. Furthermore, T1 plays an important role in synaptic transmission, although the mechanism is not understood. Since T1 homodimer has not yet been observed in neurons, further investigation is needed. In the astrocyte, which is shown in a gray square, T1 is a major isoform of TrkB receptors. The binding of BDNF to  T1 induces T1 homodimer, which results in the release of Rho GDI1 and the morphological changes of astrocytes. Moreover, T1 is involved in a Ca^2+^ influx in astrocytes. On the other hand, TTIP (truncated TrkB-interacting protein) is a binding protein of T1, but it is not clear whether it transduces the signals.

In the early developmental stage of the monkey neocortex, when T1 is not expressed, at embryonic day 120 (E120), TK+/TK+ homodimer is formed in a ligand-dependent manner [100], suggesting that the signaling pathway of TK+ mainly works during this period. Then, the adaptor proteins that interacted with TK+ are activated by the TrkB ligands (BDNF, NT-3, and NT4/5). The main signaling pathways, including the PLC-γ1, Ras/MAP, and PI3K pathways, function in neuronal plasticity, neurite outgrowth and survival, and cell motility, respectively (Fig. **2**) [59,106]. The formation of TK+ homodimer is consistent with the finding that these phenomena occur actively during the early developmental period. In the early phase of development, the expression of BDNF is at a very low level, while the other TrkB ligand, NT-3, is expressed at a relatively higher level than its expression level at the adult stage [90]. Together with the fact that NT-3 can induce the dimerization of TrkB [100], the NT-3-TK+ signaling pathway might play an important role in the regulation of the cell cycle and migration [44,122].

TK+/TK+ and T1/T1 homodimers are formed at the newborn stage (NB) of the monkey neocortex [100]. The number of axons in the corpus callosum and the anterior commissure has been reported to reach a maximum in NB and to decrease to about 75% by P60 [51,52,75,76]. The increase in T1 expression correlates well with the period when commissural axons are eliminated and synaptogenesis occurs [51,52,75,76,105]. This result suggests that T1 might be involved in the elimination of axons. As possible mechanisms, the followings may be considered: 1) the expression of T1 increases in neighbouring glial cells and T1 in glial cells absorbs the excess BDNF for axonal pruning, 2) in neurons, the increase of T1 induced the inhibition of the action of TK+ by the dominant effect of T1.

In the monkey neocortex, the density of synapses increases after birth, reaches the highest level between postnatal 2–4 months in all cortical areas, and decreases to about half of the maximum level within several years after birth [51,52,105]. The expression of T1 increases remarkably after birth [2,70,99]. Interestingly, the dendritic filopodia, which are known precursors of synaptic spines, are induced by overexpression of T1 in hippocampal neurons from postnatal rats [49]. This outgrowth of dendritic filopodia is not observed in TK+-overexpressing neurons. Thus, T1 by itself might be involved in synaptogenesis, although the mechanism is unclear.

As described above, T1 participates in axon elimination, whereas it exhibits an increase in the number of dendritic filopodia. This is a contradiction, but it may be explained by the difference in intracellular localization of TK+ and T1, such as dendrites and axons. In fact, in adult brains, T1 is concentrated in the presynaptic site [7,103], whereas TK+ is localized in both pre- and postsynaptic regions [7,103,112]. In the developing brains, the distributions of TK+ and T1 might be dynamically changed.

At the adult stage, TK-/TK- homodimer and the TK+/TK- heterodimer have been observed to form in the monkey cerebral cortex [100]. Furthermore, surprisingly, TK+ homodimer is not formed at adulthood. Although it would be very interesting to determine whether the TK+/TK-heterodimer can transduce the intracellular signals, T1 may function as a dominant negative receptor of TK+ in neurons. At the same time, T1 plays an important role in glial cells, which we discuss in the following section.

## SIGNALING PATHWAY OF T1

T1 had been hypothesized to be a dominant-negative form of TK+ because of a lack of the tyrosine kinase domain and to be involved in negative functions against TK+, such as TK+ phosphorylation [70], calcium efflux [31], neurite outgrowth [42], cell survival activity [47], and gene expression by BDNF [92]. According to this hypothesis, T1 was postulated to form a homodimer or heterodimer with TK+, which prohibited TK+ signaling or limited the availability of BDNF to neurons by trapping excess BDNF [17]. In contrast, there were several reports that provided evidence against the hypothesis that T1 was a dominant-negative form of TK+. For example, several researchers showed that the expression of T1 increases markedly at various important periods in the developing mammalian CNS, such as axonal remodeling and synaptogenesis [2,41,99,100]. The specific alignment of the intracellular domain of T1 is completely identical among mice, rats, and humans [67,86,118], suggesting that this alignment plays a unique role. In addition, T1 is capable of binding to BDNF at the same level as does TK+ [17]. Taken together with the fact that T1 has been reported to mediate signal transduction (i.e., the acid metabolite release from cells) [11], these findings raised the possibility that T1 has its own signaling pathway.

Recently, T1 has been reported to possess a signaling pathway (Fig. **2**) [96,98]. T1 is directly bound to Rho GDI1, a Rho guanine nucleotide dissociation inhibitor that can stabilize the inactive, GDP-bound form of Rho GTPase [98]. The Rho signaling pathway controls the remodeling of microfilaments, intermediate filaments, and microtubules [35, 121]. In the BDNF-T1 signaling pathway, Rho GDI1 is released from T1 in a BDNF-dependent manner, which causes decreases in the activities of Rho-signaling molecules such as RhoA, Rho-associated kinase (ROCK), p21-activated kinase (PAK), and extracellular-signal regulated kinase (ERK) 1/2 [96]. Consequently, T1 alters the cell morphology of astrocytes in primary cultures and acute slices [93,98].

T1 has been involved in the intracellular Ca^2+^ influx in astrocytes, *via* PLC>IP3 production [109]. Since the PLC pathway plays an important role on neuronal plasticity [119], T1 in neurons might participate in this process.

Another binding protein of T1 is truncated TrkB-interacting protein (TTIP), which is isolated from 15N neuroblastoma cells by coimmunoprecipitation with GST fusion protein containing the intracellular juxtamembrane of T1 (Fig. **2**) [73]. TTIP has a molecular weight of 61 kDa. However, BDNF stimulation cannot modulate the interaction between T1 and TTIP. It is also uncertain whether Rho GDI1 and TTIP bind directly to the different motifs in the T1-specific region or compete for the same binding site. Further studies on TTIP are needed in the future.

## REGULATION OF CELL MORPHOLOGY BY TRKB ISOFORMS

One function of the BDNF-TrkB signaling pathway is that it is heavily involved in the regulation of the cell morphology. BDNF regulates the branching and extension of axons and dendrites during development both *in vitro* and *in vivo* [4,25-27,58,63,79,81,82,84,113]. In addition, treatment with BDNF increases in the number of synapses [1,4,23, 113,116,117]. These experiments were performed using developing dissociated neurons, brain slices, and animals, suggesting that TK+ mainly functioned in neurons in these studies. In the P14 ferret neocortex, where indeed the expression of TK+ is several times that of T1 [2], BDNF administration increases the length and complexity of dendrites [82,84]. Interestingly, the laminar specificity for neurotrophin response is observed: neurons in layers 4 and 5 to BDNF and neurons in layers 5 and 6 to NT-4. In these layers that are responsible to TrkB ligands, TK+ like immunoreactivity is intensely detected at P10-24. Thus, TK+ promotes axonal and dendritic growth during development.

Studies of T1 with regard to cell morphology employed the strategy of T1 overexpression in cell line and slice cultures. In the N2a cell line, the transient overexpression of T1 led to a ligand-independent change of cell morphology, such as the growth of filopodia and processes [48]. The study demonstrated that deletion mutants lacking the T1 specific intracellular domain induce filopodia and processes, but the mutant lacking the extracellular domain failed to have this effect. In addition, p75 was not involved in this process. Thus, the authors suggested that the extracellular domain of T1 might function as a cell adhesion molecule. Another study in rat hippocampal primary cultures [49] showed that T1 induced the formation of dendritic filopodia, which occurred independent of ligand formation. In contrast, the interaction between T1 and p75 was essential for the induction of filopodia. This might have been due to material differences, such as the cell line [48] or primary cultured hippocampal neurons [49]. The study using P14 ferret neocortical slice culture showed that TK+ and T1 regulated distinct modes of dendritic growth [130]. The transfection of TK+ induced prominent outgrowth of short dendrites that extended from the cell body and the proximal region of the apical dendrites. In contrast, the transfection of T1 did not increase short dendrites near the soma, but it did elevate the arborization of distal dendrites. Providing exogenous ligands blocked the distal growth of dendrites in T1-transfected neurons. Furthermore, in proximal dendrites, the treatment with ligands decreased dendritic complexity compared with the control level. Most recently, in T1-deficient mice, morphological abnormalities in the length and complexity of neurons in the basolateral amygdala were described [21]. Considering that the expression of T1 increased at the stage of synaptogenesis, T1 might have fine-tuned the growth of dendrites, axons and synaptic structures, by the interaction with TK+. Further examination of the function of T1 in regulating neuronal morphology will be interesting.

Most importantly, T1 plays a role on astrocyte functions. For example, T1 induced a rapid change of astrocytic morphology *via* Rho GTPase in primary astrocyte cultures [98] and in the rat neocortex layer I [93]. Additionally, T1 controlled calcium entry into astrocytes [109]. Since the release of BDNF is highly regulated by neuronal activity [50,71], these findings led us to the idea that BDNF release by neuronal activities induces morphological changes of astrocytes in the CNS. Recent studies have shed light on the interactions between neurons and glial cells [38,124,128]. In particular, researchers have demonstrated that calcium entry into astrocytes modulated synaptic transmission [14,39]. In addition, astrocytic endfeet enwrap synapses [127], i.e., those synapses referred to as tripartite synapses [8]. Furthermore, astrocytic processes surrounding active synapses have been described as altering their morphology in the brainstem [56], hypothalamus [77], cerebellum [61], hippocampus [13], and neocortex [93] of infant- to pubertal-stage rodents, suggesting that the tripartite synapse is a common structure in the CNS. In contrast, alterations of fine neuronal structures such as dendrites and spines in the neocortex of adult mice hardly occur under normal conditions [45,126]. These findings suggest that the morphological alteration of astrocytes may be essential for the maintenance and plasticity of synaptic transmission, as well as for transmitter clearance. Therefore, neuronal and glial structural modifications might be regulated by the interaction of TK+ and T1 in neurons and the T1 in astrocytes, respectively.

## TRKB ISOFORMS IN THE SYNAPTIC PLASTICITY

BDNF-TrkB signaling has an effect on morphological changes of neurons and glias and plays an important role in synaptic plasticity [65,72,85,87]. In light of this, we wanted to explore two important issues, 1) activity-dependent expression and secretion of BDNF, and 2) subcellular localization of TrkB subtypes, i.e., pre- or postsynaptic sites.

Not only *in vitro* stimulations such as the administration of drugs but also physiological stimulations, such as exercise [91], visual input [22], and whisker stimulation [107], showed the increase of BDNF expression and secretion. It is unclear whether dendritic production of BDNF (i.e., BDNF mRNA targeting to dendrites) and concentration of BDNF protein in the secretion vesicles occur in the active synapse [51,72]. Furthermore, it has not yet been clarified whether BDNF is released from the vesicles in the pre- or postsynaptic sites [3,36,37,50,71], like neuropeptide transmitters [111].

TrkB subtypes are widely distributed throughout the brain, as described in the previous section. However, considering that the signaling pathway of TK+ is distinct from that of T1, it is important to clarify the subcellular localization of TK+ and T1 in neurons. Subcellular fractionation of the rat brain showed that 1) both TrkB subtypes were concentrated in synaptic membrane fraction [7,94,97,103], 2) TK+ and T1 exhibited a differential subcellular distribution; TK+ was present in the presynaptic active zone and postsynaptic density, while T1 was mainly distributed in the presynaptic active zone [7,103]. Interestingly, using cultured hippocampal neurons infected with the T1-expressing adenovirus vector, Schuman’s group demonstrated that presynaptic, but not postsynaptic, expression of T1 inhibited BDNF enhancement of synaptic transmission, whereas activation of TrkB-associated signaling enhanced neurotransmitter release from presynaptic terminals [78]. Although pre- and postsynaptic modifications are involved in long-term potentiation, at least presynaptic T1 might play an important role in the regulation of initial synaptic potentiation between neurons. Since T1 inhibits the phosphorylation of TK+, the activation of BDNF-TK+ signaling may be required for BDNF-induced potentiation. On the other hand, Rho GTPases are involved in Ca^2+^-dependent neurotransmitter exocytosis *via* the regulation of actin filament [30,88]. Thus, the BDNF-T1-Rho GDI1 signaling cascade may regulate the neurotransmitter release, regulating Rho GTPases activity.

## PHARMACOLOGICAL USEFULNESS OF T1 AS A MOLECULAR SWITCH

The T1 signaling cascade challenges the conventional view that T1 acts as a dominant negative form of TK+. It is reasonable to assume that T1 could exert dual roles in an age-dependent manner and/or by subcellular and cellular localization. In neurons, T1 could act as a dominant negative form of TK+ through the formation of the TK+/TK- heterodimer in adulthood. In astrocytes, T1 could act as a negative regulator for the Rho signaling cascade. Thus, T1 may be a Janus-faced receptor of BDNF as a “molecular switch.” Also, the change of TrkB receptor dimerization may be one of the mechanisms generating the variety of biological functions of BDNF during development.

If we can control each expression of TrkB subtypes in a certain cell type, i.e., in a neuron- or astrocyte-specific manner, by drugs or gene-transferring treatment in the near future, the results might be useful in the treatment for psychiatric and neurological diseases, including depression and suicide [34], schizophrenia [104], and neurodegenerative diseases [29]. These studies suggest that it is essential for maintaining neuronal functions to regulate adequately the expression of T1. For example, the decrease in expression of T1 is observed in the frontal cortex of suicide completers [34]. Interestingly, the methylation in the trkB promoter regions is significantly reduced, which results in only the decrease in T1 expression without the change of TK+ expression [34]. In the model mouse of schizophrenia, both mRNA and protein levels of T1 were significantly higher in the frontal cortex, but those of TK+ were not altered [104]. Furthermore, using a trisomic mouse model, the suitable expression level of T1 is important for the survival of neocortical and hippocampal neurons. Taken together, pharmaceutical preparations to regulate the proper expression of T1, such as T1 siRNA and cDNA [93,98] and synthetic peptide of T1 specific C-terminal sequence [98], will be valuable for the treatment of psychiatric and neurological disorders. In addition, the combination use of T1 siRNA and cDNA and cell type-specific promoters can be more useful.

T1 has been shown to be expressed in the neurogenic regions [43,123]. Recent study suggests that overexpression of T1 increases the proliferation of neural progenitor cells [123]. Interestingly, BDNF has anti-proliferative activity on the self-renewal of neural stem cells; however, it also functions as a differentiation factor for stem cells, which are affected by the expression of TK+. Thus, the rate of TrkB subtype expression in stem cells is of importance in determining the balance between proliferation and differentiation. *In vivo* study also showed that dopaminergic periglomerular interneurons in the olfactory bulb were decreased in TrkB KO mice. Moreover, calbindin-positive cells were slightly decreased, compared with the control, suggesting that TrkB may play a selective role in regulating the proliferation and differentiation of subtypes of specific interneurons [43]. Furthermore, as described in the above sections, TrkB subtypes influence neural plasticity *via* regulation of the neuronal and glial morphology. Therefore, the control of BDNF-TrkB signaling can regenerate neurons and repair neuronal networks as therapy following a brain injury such as trauma or ischemia.

## References

[1] Aguado F, Carmona MA, Pozas E, Aguiló A, Matrínes-Guijarro FJ, Alcantara S, Borrell V, Yuste R, Ibañes CF, Soriano E (2003). BDNF Regulates Spontaneous Correlated Activity at Early Developmental Stages by Increasing Synaptogenesis and Expression of the K^+^/Cl^-^ Co-transporter KCC2. Development.

[2] Allendoerfer KL, Cabelli RJ, Escanòn E, Kaplan DR, Nikolics K, Shatz CJ (1994). Regulation of Neurotrophin Receptors During the Maturation of the Mammalian Visual System. J. Neurosci.

[3] Aloyz R, Fawcett JP, Kaplan DR, Murphy RA, Miller FD (1999). Activity-dependent Activation of TrkB Neurotrophin Receptors in the Adult CNS. Learn. Mem.

[4] Alsina B, Vu T, Cohen-Cory S (2001). Visualizing Synapse Formation in Arborizing Optic Axons *in vivo*: Dynamics and Modulation by BDNF. Nat. Neurosci.

[5] Alter CA, Cai N, Bliven T, Juhansz M, Conner JM, Acheson AL, Lindsay RM, Wiegand SJ (1997). Anterograde Transport of Brain-derived Neurotrophic Factor and Its Role in the Brain. Nature.

[6] Anderson KD, Alderson RF, Alter CA, Distefano PS, Corcoran TL, Lindsay RM, Wiegand SJ (1995). Differential Distribution of Exogenous BDNF, NGF, and NT-3 in the Brain Corresponds to the Relative Abundance and Distribution of High-affinity and Low-affinity Neurotrophin Receptors. J. Comp. Neurol.

[7] Aoki C, Wu K, Elste A, Len G, Lin S, McAuliffe G, Black IB (2000). Localization of Brain-derived Neurotrophic Factor and TrkB Receptors to Postsynaptic Densities of Adult Rat Cerebral Cortex. J. Neurosci. Res.

[8] Araque A, Parpura V, Sanzgiri RP, Haydon PG (2001). Tripartite Synapses: Glia, the Unacknowledged Partner. Trends Neurosci.

[9] Armanini MP, McMahon SB, Sutherland J, Shelton DL, Phillips HS (1995). Truncated and Catalytic Isoforms of TrkB are Co-expressed in Neurons of Rat and Mouse CNS. Eur. J. Neurosci.

[10] Barbacid M (1994). The Trk Family of Neurotrophin Receptors. J. Neurobiol.

[11] Baxter GT, Radeke MJ, Kuo RC, Makrides V, Hinkle B, Hoang R, Medina-Selby A, Coit D, Valenzuela P, Feinstein SC (1997). Signal Transduction Mediated by the Truncated TrkB Receptor Isoforms, TrkB.T1 and TrkB.T2. J. Neurosci.

[12] Beck KD, Lamballe F, Klein R, Barbacid M, Schauwecker PE, McNeill TH, Finch CE, Hefti F, Day JR (1993). Induction of Noncatalytic TrkB Neurotrophin Receptor During Axonal Sprouting in the Adult Hippocampus. J. Neurosci.

[13] Benediktsson AM, Schachtele SJ, Green SH, Dailey ME (2005). Ballistic Labeling and Dynamic Imaging of Astrocytes in Organotypic Hippocampal Slice Cultures. J. Neurosci. Methods.

[14] Bezzi P, Gundersen V, Galbete JL, Seifert G, Steinhauser C, Pilati E, Volterra A (2004). Astrocytes Contain a Vesicular Compartment That is Competent for Regulated Exocytosis of Glutamate. Nat. Neurosci.

[15] Bibel M, Barde YA (2000). Neurotrophins: Key Regulators of Cell Fate and Cell Shapes in the Vertebrate Nervous System. Genes Dev.

[16] Bibel M, Hoppe E, Barde YA (1999). Biochemical and Functional Interactions Between the Neurotrophin Receptors *trk* and p75^NTR^. EMBO J.

[17] Biffo S, Offenhäuser N, Carter BD, Barde YA (1995). Selective Binding and Internalization by Truncated Receptors Restrict the Availability of BDNF During Development. Development.

[18] Bothwell M (1995). Functional Interactions of Neurotrophins and Neurotrophin Receptors. Annu. Rev. Neurosci.

[19] Bramham CR, Messaoudi E (2005). BDNF Function in Adult Synaptic Plasticity: the Synaptic Consolidation Hypothesis. Prog. Neurobiol.

[20] Cabelli RJ, Allendoerfer KL, Radeke MJ, Welcher AA, Feinstein SC, Shatz CJ (1996). Changing Patterns of Expression and Subcellular Localization of TrkB in the Developing Visual System. J. Neurosci.

[21] Carim-Todd L, Bath KG, Fulgenzi G, Yanpallewar S, Jing D, Barrick CA, Becker J, Buckley H, Dorsey SG, Lee FS, Tessarollo L (2009). Endogenous Truncated TrkB.T1 Receptor Regulates Neuronal Complexity and TrkB Kinase Receptor Function *In Vivo*. J. Neuosci.

[22] Castrén E, Zafra F, Thoenen H, Lindholm D (1992). Light Regulates Expression of Brain-derived Neurotrophic Factor mRNA in Rat Visual Cortex. Proc. Natl. Acad. Sci. USA.

[23] Causing CG, Gloster A, Aloyz R, Bamji SX, Chang E, Fawcett J, Kuchel G, Miller FD (1997). Synaptic Innervation Density is Regulated by Neuron-derived BDNF. Neuron.

[24] Chao MV (2003). Neurotrophins and Their Receptors: a Convergence Point for Many Signaling Pathways. Nat. Rev. Neurosci.

[25] Cohen-Cory S (1996). BDNF Modulates, but does not Mediate, Activity-dependent Branching and Remodeling of Optic Axon Arbors *in vivo*. J. Neurosci.

[26] Cohen-Cory S, Fraser SE (1995). Effects of Brain-derived Neurotrophic Factor on Optic Axon Branching and Remodelling *In Vivo*. Nature.

[27] Danzer SC, Crooks KRC, Lo DC, McNamara JO (2002). Increased Expression of Brain-derived Neurotrophic Factor Induces Formation of Basal Dendrites and Axonal Branching in Dentate Granule Cells in Hippocampal Explant Cultures. J. Neurosci.

[28] Dechant G, Barde YA (2002). The Neurotrophin Receptor p75 (NTR): Novel Functions and Implications for Diseases of the Nervous System. Nat. Neurosci.

[29] Dorsey SG, Renn CL, Carim-Todd L, Barrick CA, Bambrick L, Krueger BK, Ward CW, Tessarollo L (2006). *In Vivo* Restoration of Physiological Levels of Truncated TrkB.T1 Receptor Rescues Neuronal Cell Death in a Trisomic Mouse Model. Neuron.

[30] Doussau F, Gasman S, Humeau Y, Vitiello F, Popoff M, Boquet P, Bader MF, Poulain B (2000). A Rho-related GTPase is Involved in Ca^2+^-dependent Neurotransmitter Exocytosis. J. Biol. Chem.

[31] Eide FF, Vining ER, Eide BL, Zang K, Wang X, Reichardt LF (1996). Naturally Occurring Truncated TrkB Receptors Have Dominant Inhibitory Effects on Brain-derived Neurotrophic Factor Signaling. J. Neurosci.

[32] Edwards DP (2005). Regulation of Signal Transduction Pathways by Estrogen and Progesteron. Annu. Rev. Physiol.

[33] Ernfors P, Wetmore C, Olson L, Persson H (1990). Identification of Cells in Rat Brain and Peripheral Tissues Expressing mRNA for Members of the Nerve Growth Factor Family. Neuron.

[34] Ernst C, Deleva V, Deng X, Sequeira A, Pomarenski A, Klempan T, Ernst N, Quirion R, Gratton A, Szyf M, Turecki G (2009). Alternative Splicing, Methylation State, and Expression Prolife of Tropomyosin-related Kinase B in the Frontal Cortex of Suicide Completers. Arch. Gen. Psychiatry.

[35] Etienne-Manneville S, Hall A (2002). Rho GTPases in Cell Biology. Nature.

[36] Fawcett JP, Aloyz R, McLean JH, Pareek S, Miller FD, McPherson PS, Murphy RA (1997). Detection of Brain-derived Neurotrophic Factor in a Vesicular Fraction of Brain Synaptosomes. J. Biol. Chem.

[37] Fawcett JP, Bamji SX, Causing CG, Aloyz R, Ase AR, Reader TA, McLean JH, Miller FD (1998). Functional Evidence that BDNF is an Anterograde Neuronal Trophic Factor in the CNS. J. Neurosci.

[38] Fellin T, Carmignoto G (2004). Neuron-to-astrocyte Signalling in the Brain Represents a Distinct Multifunctional unit. J. Physiol.

[39] Fiacco TA, McCarthy KD (2004). Intracellular Astrocyte Calcium Waves *In Situ* Increase the Frequency of Spontaneous AMPA Receptor Currents in CA1 Pyramidal Neurons. J. Neurosci.

[40] Frisén J, Verge VMK, Fried K, Risling M, Persson H, Trotter J, Hökfelt T, Lindholm D (1993). Characterization of Glial TrkB Receptors: Differential Response to Injury in the Central and Peripheral Nervous Systems. Proc. Natl. Acad. Sci. USA.

[41] Fryer RH, Kaplan DR, Feinstein SC, Radeke MJ, Grayson DR, Kromer LF (1996). Developmental and Mature Expression of Full-length and Truncated TrkB Receptors in the Rat Forebrain. J. Comp. Neurol.

[42] Fryer RH, Kaplan DR, Kromer LF (1997). Truncated TrkB Receptors on Nonneuronal Cells Inhibit BDNF-induced Neurite Outgrowth *In Vitro*. Exp. Neurol.

[43] Galvão RP, Garcia-Verdugo JM, Alvarez-Buylla A (2008). Brain-derived Neurotrophic Factor Signaling does not Stimulate Subventricular Zone Neurogenesis in Adult Mice and Rats. J. Neurosci.

[44] Ghosh A, Greenberg ME (1995). Distinct Roles for bFGF and NT-3 in the Regulation of Cortical Neurogenesis. Neuron.

[45] Graveley BR (2001). Alternative Splicing: Increasing Diversity in the Proteomic World. Trends Genetics.

[46] Grutzendler J, Kasthuri N, Gan WB (2002). Long-term Dendritic Spine Stability in the Adult Cortex. Nature.

[47] Haapasalo A, Koponen E, Hoppe E, Wong G, Castrén E (2001). Truncated TrkB.T1 is Dominant Negative Inhibitor of TrkB.TK+-mediated Cell Survival. Biochem. Biophys. Res. Commun.

[48] Haapasalo A, Saarelainen T, Moshnyakov M, Arumäe U, Kiema TR, Saarma M, Wong G, Castrén E (1999). Expression of the Naturally Occurring Truncated TrkB Neurotrophin Receptor Induces Outgrowth of Filopodia and Processes in Neuroblastoma Cells. Oncogene.

[49] Hartmann M, Brigadski T, Erdmann KS, Holtmann B, Sendtner M, Narz F, Lessmann V (2004). Truncated TrkB Receptor-induced Outgrowth of Dendritic Filopodia Involves the p75 Neurotrophin Receptor. J. Cell Sci.

[50] Hartmann M, Heumann R, Lessmann V (2001). Synaptic Secretion of BDNF After High-frequency Stimulation of Glutamatergic Synapses. EMBO J.

[51] Hayashi M (1992). Ontogeny of Some Neuropeptides in the Primate Brain. Prog. Neurobiol.

[52] Hayashi M (2008). Neuroactive Molecules in the Brains of Non-human Primates and Their Therapeutic Application to Neurodegenerative Disorders. Cent. Nerv. Syst. Agents Med. Chem.

[53] Hayashi M, Mitsunaga F, Itoh M, Shimizu K, Yamashita A (2000). Development of Full-length Trk B-Immunoreactive Structures in the Prefrontal and Visual Cortices of the Macaque Monkey. Anat. Embryol.

[54] Hayashi M, Mitsunaga F, Ohira K, Shimizu K, Yamashita A (1999). Development of Full-length Trk B- Immunoreactive Structures in the Hippocampal Formation of the Macaque Monkey. Anat. Embryol.

[55] Hayashi M, Mitsunaga F, Ohira K, Shimizu K (2001). Changes in BDNF-immunoreactive Structures in the Hippocampal Formation of the Aged Macaque Monkey. Brain Res.

[56] Hirrlinger J, Hülsmann S, Kirchhoff F (2004). Astroglial Processes Show Spontaneous Motility at Active Synaptic Terminals *In Situ*. Eur. J. Neurosci.

[57] Hofer M, Pagliusi SR, Hohn A, Leibrock J, Barde YA (1990). Regional Distribution of Brain-derived Neurotrophic Factor mRNA in the Adult Mouse Brain. EMBO J.

[58] Hu B, Nikolakopoulou AM, Cohen-Cory S (2005). BDNF Stabilizes Synapses and Maintains the Structural Complexity of Optic Axons *In Vivo*. Development.

[59] Huang EJ, Reichardt LF (2003). Trk Receptors: Roles in Neuronal Signal Transduction. Annu. Rev. Biochem.

[60] Huntley GW, Benson DL, Jones EG, Isackson PJ (1992). Developmental Expression of Brain-derived Neurotrophic Factor mRNA by Neurons of Fetal and Adult Monkey Prefrontal Cortex. Dev. Brain Res.

[61] Iino M, Goto K, Kakegawa W, Okado H, Sudo M, Ishiuchi S, Miwa A, Takayasu Y, Saito I, Tsuzuki K, Ozawa S (2001). Glia-synapse Interaction Through Ca^2+^-permeable AMPA Receptors in Bergmann Glia. Science.

[62] Isackson PJ, Huntsman MM, Murray KD, Gall CM (1991). BDNF mRNA Expression is Increased in Adult Rat Forebrain After Limbic Seizures: Temporal Patterns of Induction Distinct from NGF. Neuron.

[63] Jin X, Hu H, Mathers PH, Agmon A (2003). Brain-derived Neurotrophic Factor Mediates Activity-dependent Dendritic Growth in Nonpyramidal Neocortical Interneurons in Developing Organotypic Cultures. J. Neurosci.

[64] Kang H, Schuman EM (1996). A Requirement for Local Protein Synthesis in Neurotrophin-induced Hippocampal Synaptic Plasticity. Science.

[65] Kang H, Welcher AA, Shelton D, Schuman EM (1997). Neurotrophins and Time: Different Roles for TrkB Signaling in Hippocampus Long-term Potentiation. Neuron.

[66] Kaplan DR, Miller FD (2000). Neurotrophin Signal Transduction in the Nervous System. Curr. Opin. Neurobiol.

[67] Klein R, Conway D, Parada LF, Barbacid M (1990). The TrkB Tyrosine Kinase Gene Codes for a Second Neurogenic Receptor that Lacks the Catalytic Kinase Domain. Cell.

[68] Klein R, Martin-Zanca D, Barbacid M, Parada LF (1990). Expression of the Tyrosine Kinase Receptor Gene TrkB is Confined to the Murine Embryonic and Adult Nervous System. Development.

[69] Klein R, Parada LF, Coulier F, Barbacid M (1989). TrkB, a Novel Tyrosine Protein Kinase Receptor Expressed During Mouse Neural Development. EMBO J.

[70] Knüsel B, Rabin SJ, Hefti F, Kaplan DR (1994). Regulated Neurotrophin Receptor Responsiveness During Neuronal Migration and Early Differentiation. J. Neurosci.

[71] Kohara K, Kitamura A, Morishima M, Tsumoto T (2001). Activity-dependent Transfer of Brain-derived Neurotrophic Factor to Postsynaptic Neurons. Science.

[72] Kovalchuk Y, Hanse E, Kafitz KW, Konnerth A (2002). Postsynaptic Induction of BDNF-mediated Long-term Potentiation. Science.

[73] Kryl D, Barker PA (2000). TTIP is a Novel Protein That Interacts With the Truncated T1 TrkB Neurotrophin Receptor. Biochem. Biophys. Res. Commun.

[74] Kumanogoh H, Asami J, Nakamura S, Inoue T (2008). Balanced Expression of Various TrkB Receptor Isoforms from the Ntrk2 Gene Locus in the Mouse Nervous System. Mol. Cell. Neurosci.

[75] LaMantia AS, Rakic P (1990). Axon Overproduction and Elimination in the Corpus Callosum of the Developing Rhesus Monkey. J. Neurosci.

[76] LaMantia AS, Rakic P (1994). Axon Overproduction and Elimination in the Anterior Commissure of the Developing Rhesus Monkey. J. Comp. Neurol.

[77] Langle SL, Poulain DA, Theodosis DT (2003). Induction of Rapid, Activity-dependent Neuronal-glial Remodeling in the Adult Rat Hypothalamus *In Vitro*. Eur. J. Neurosci.

[78] Li Y, Xu Y, Ju D, Lester HA, Davidson N, Schuman EM (1998). Expression of a Dominant Negative TrkB Receptor, T1, Reveals a Requirement for Presynaptic Signaling in BDNF-induced Synaptic Potentiation in Cultured Hippocampal Neurons. Proc. Natl. Acad. Sci. USA.

[79] Lom B, Cohen-Cory S (1999). Brain-derived Neurotrophic Factor Differentially Regulates Retinal Ganglion Cell Dendritic and Axonal Arborization *In Vivo*. J. Neurosci.

[80] Marinissen MJ, Gutkind JS (2001). G-Protein-Coupled Receptors and Signaling Networks: Emerging Paradigms. Trends Pharmacol. Sci.

[81] Marshak S, Nikolakopoulou AM, Dirks R, Martens GJ, Cohen-Cory S (2007). Cell-Autonomous TrkB Signaling in Presynaptic Retinal Ganglion Cells Mediates Axon Arbor Growth and Synapse Maturation During the Establishment of Retinotectal Synaptic Connectivity. J. Neurosci.

[82] McAllister AK, Katz LC, Lo DC (1996). Neurotrophin Regulation of Cortical Dendritic Growth Requires Activity. Neuron.

[83] McAllister AK, Katz LC, Lo DC (1999). Neurotrophins and Synaptic Plasticity. Annu. Rev. Neurosci.

[84] McAllister AK, Lo DC, Katz LC (1995). Neurotrophins Regulate Dendritic Growth in Developing Visual Cortex. Neuron.

[85] Messaoudi E, Ying S, Kanhema T, Croll SD, Bramham CR (2002). Brain-derived Neurotrophic Factor Triggers Transcription-dependent, Late Phase Long-term Potentiation *In Vivo*. J. Neurosci.

[86] Middlemas DS, Lindberg RA, Hunter T (1991). TrkB, a Neural Receptor Protein-tyrosine Kinase: Evidence for a Full-length and Two Truncated Receptors. Mol. Cell. Biol.

[87] Minichiello L, Calella AM, Medina DL, Bonhoeffer T, Klein R, Korte M (2002). Mechanism of TrkB-mediated Hippocampal Long-term Potentiation. Neuron.

[88] Morales M, Colicos MA, Goda Y (2000). Actin-dependent Regulation of Neurotransmitter Release at Central Synapses. Neuron.

[89] Mori T, Shimizu K, Hayashi M (2004). Differential Expression Patterns of TrkB Ligands in the Macaque Monkey Brain. Neuroreport.

[90] Mori T, Takumi K, Shimizu K, Oishi T, Hayashi M (2006). Heterogeneity of the Developmental Patterns of Neurotrophin Protein Levels Among Neocortical Areas of Macaque Monkeys. Exp. Brain Res.

[91] Neeper SA, Gómez-Pinilla F, Choi J, Cotman CW (1996). Physical Activity Increases mRNA for Brain-derived Neurotrophic Factor and Nerve Growth Factor in Rat Brain. Brain Res.

[92] Offenhäuser N, Muzio V, Biffo S (2002). BDNF Binding to Truncated trkB.T1 does not Affect Gene Expression. Neuroreport.

[93] Ohira K, Funatsu N, Homma KJ, Sahara Y, Hayashi M, Kaneko T, Nakamura S (2007). Truncated TrkB-T1 Regulates the Morphology of Neocortical Layer I Astrocytes in Adult Rat Brain Slices. Eur. J. Neurosci.

[94] Ohira K, Funatsu N, Nakamura S, Hayashi M (2004). Expression of BDNF and TrkB Receptor Subtypes in the Postnatal Developing Purkinje Cells of Monkey Cerebellum. Gene Expr. Patterns.

[95] Ohira K, Hayashi M (2003). Expression of TrkB Subtypes in the Adult Monkey Cerebellar Cortex. J. Chem. Neuroanat.

[96] Ohira K, Homma KJ, Hirai H, Nakamura S, Hayashi M (2006). TrkB-T1 Regulates the RhoA Signaling and Actin Cytoskeleton in Glioma Cells. Biochem. Biophys. Res. Commun.

[97] Ohira K, Maekawa S, Hayashi M (2000). Absence of TrkB and Insulin Receptor β in the Triton Insoluble Low-density Fraction (raft). Neuroreport.

[98] Ohira K, Kumanogoh H, Sahara Y, Homma KJ, Hirai H, Nakamura S, Hayashi M (2005a). A Truncated Tropo-myosine-related Kinase B Receptor, T1, Regulates Glial Cell Morphology *via* Rho GDP Dissociation Inhibitor 1. J. Neurosci.

[99] Ohira K, Shimizu K, Hayashi M (1999). Change of Expression of Full-length and Truncated TrkBs in the Developing Monkey Central Nervous System. Dev. Brain Res.

[100] Ohira K, Shimizu K, Hayashi M (2001). TrkB Dimerization During Development of the Prefrontal Cortex of the Macaque. J. Neurosci. Res.

[101] Ohira K, Shimizu K, Yamashita A, Hayashi M (2005b). Differential Expression of the Truncated TrkB Receptor, T1, in the Primary Motor and Prefrontal Cortices of the Adult Macaque Monkey. Neurosci. Lett.

[102] Okuno H, Tokuyama W, Li YX, Hashimoto T, Miyashita Y (1999). Quantitative Evaluation of Neurotrophin and *trk* mRNA Expression in Visual and Limbic Areas Along the Occipito-temporo-hippocampal Pathway in Adult Macaque monkeys. J. Comp. Neurol.

[103] Pereira DB, Rebola N, Rodrigues RJ, Cunha RA, Carvalho AP, Duarte CB (2006). TrkB Receptors Modulation of Glutamate Release is Limited to a Subset of Nerve Terminals in the Adult Rat Hippocampus. J. Neurosci. Res.

[104] Pillai A, Mahadik SP (2008). Increased Truncated TrkB Receptor Expression and Decreased BDNF/TrkB Signaling in the Frontal Cortex of Reeler Mouse Model of Schizophrenia. Schizophr. Res.

[105] Rakic P, Bourgeois JP, Eckenhoff MF, Zecevic N, Goldman-Rakic PS (1986). Concurrent Overproduction of Synapses in Diverse Regions of the Primate Cerebral Cortex. Science.

[106] Reichardt LF (2006). Neurotrophin-regulated Signalling Pathways. Philos. Trans. R. Soc. B.

[107] Rocamora N, Welker E, Pascual M, Soriano E (1996). Upregulation of BDNF mRNA Expression in the Barrel Cortex of Adult Mice After Sensory Stimulation. J. Neurosci.

[108] Romanczyk TB, Weickert CS, Webster MJ, Herman MM, Akil M, Kleinman JE (2002). Alterations in trkB mRNA in the Human Prefrontal Cortex Throughout the Lifespan. Eur. J. Neurosci.

[109] Rose CR, Blum R, Pichler B, Lepier A, Kafitz KW, Konnerth A (2003). Truncated TrkB-T1 Mediates Neurotrophin-evoked Calcium Signalling in Glia Cells. Nature.

[110] Roux. PP, Barker PA (2002). Neurotrophin Signaling Through the p75 Neurotrophin Receptor. Prog. Neurobiol.

[111] Salio C, Averill S, Priestley JV, Merighi A (2007). Costorage of BDNF and Neuropeptides Within Individual Dence-core Vesicles in Central and Peripheral Neurons. Dev. Neurobiol.

[112] Salio C, Lossi L, Ferrini F, Merighi A (2005). Ultrastructural Evidence for a Pre- and Postsynaptic Localization of Full-length TrkB Receptors in Substantia Gelatinosa (Lamina II) of Rat and Mouse Spinal Cord. Eur. J. Neurosci.

[113] Sanchez AL, Matthews BJ, Meynard MM, Hu B, Javed S, Cohen-Cory S (2006). BDNF Increases Synapse Density in Dendrites of Developing Tectal Neurons *In Vivo*. Development.

[114] Schoups AA, Elliott RC, Friedman WJ, Black IB (1995). NGF and BDNF are Differentially Modulated by Visual Experience in the Developing Geniculocortical Pathway. Dev. Brain Res.

[115] Segal RA (2003). Selectivity in Neurotrophin Signaling: Theme and Variations. Annu. Rev. Neurosci.

[116] Seil FJ (1999). BDNF and NT-4, but not NT-3, Promote Development of Inhibitory Synapses in the Absence of Neuronal Activity. Brain Res.

[117] Seil FJ, Drake-Baumann R (2000). TrkB Receptor Ligands Promote Activity-dependent Inhibitory Synaptogenesis. J. Neurosci.

[118] Shelton DL, Sutherland J, Gripp J, Camerato T, Armanini MP, Phillips HS, Carroll K, Spencer SD, Levinson AD (1995). Human Trks: Molecular Cloning, Tissue Distribution, and Expression of Extracellular Domain Immunoadhesins. J. Neurosci.

[119] Sheng M, Kim MJ (2002). Postsynaptic Signaling and Plasticity Mechanisms. Science.

[120] Stamm S, Ben-Ari S, Rafalska I, Tang Y, Zhang Z, Toiber D, Thanaraj TA, Soreq H (2005). Function of Alternative Splicing. Gene.

[121] Takai Y, Sasaki T, Matozaki T (2001). Small GTP-binding Proteins. Physiol. Rev.

[122] Takumi K, Mori T, Shimizu K, Hayashi M (2005). Developmental Changes in Concentrations and Distributions of Neurotrophins in the Monkey Cerebellar Cortex. J. Chem. Neuroanat.

[123] Tervonen TA, Ajamian F, De Wit J, Verhaagen J, Castrén E, Castrén M (2006). Overexpression of a Truncated TrkB Isoform Increases the Proliferation of Neural Progenitors. Eur. J. Neurosci.

[124] Theodosis DT, Poulain DA, Oliet SHR (2008). Activity-dependent Structural and Functional Plasticity of Astrocyte-neuron Interactions. Physiol. Rev.

[125] Thoenen H (2000). Neurotrophins and Activity-dependent Plasticity. Prog. Brain Res.

[126] Trachtenberg JT, Chen BE, Knott GW, Feng G, Sanes JR, Welker E, Svoboda K (2002). Long-term *In Vivo* Imaging of Experience-dependent Synaptic Plasticity in Adult Cortex. Nature.

[127] Ventura R, Harris KM (1999). Three-dimensional relationships between hippocampal synapses and astrocytes. J. Neurosci.

[128] Volterra A, Meldolest J (2005). Astrocytes, From Brain Glue to Communication Elements: the Revolution Continues. Nat. Rev. Neurosci.

[129] Webster MJ, Herman MM, Kleinman JE, Weickert CS (2006). BDNF and TrkB mRNA Expression in the Hippocampus and Temporal Cortex During the Human Lifespan. Gene Expr. Patterns.

[130] Yacoubian TA, Lo DC (2000). Truncated and Full-length TrkB Receptors Regulate Distinct Modes of Dendritic Growth. Nat. Neurosci.

[131] Yan Q, Radeke ML, Matheson CR, Talvenheimo J, Welcher AA, Feinstein SC (1997). Immunocytochemical Localization of TrkB in the Central Nervous System of the Adult Rat. J. Comp. Neurol.

[132] Yan Q, Rosenfeld RD, Matheson CR, Hawkins N, Lopez OT, Bennett L, Welcher AA (1997). Expression of Brain-derived Neurotrophic Factor Protein in the Adult Rat Central Nervous System. Neuroscience.

[133] Zhang H, Li L, Zou X, Song X, Hu Y, Feng Z, Wang TT (2007). Immunohistochemical Distribution of NGF, BDNF, NT-3, NT-4 in Adult Rhesus Monkey Brains. J. Histochem. Cytochem.

